# Psychopathologies in post-COVID outpatients differ from a psychosomatic control sample in a cross-sectional study

**DOI:** 10.1007/s44192-026-00475-8

**Published:** 2026-05-20

**Authors:** Gloria-Beatrice Wintermann, Malte Hahnfeld, Maria Lenk, Amalia Hanßke, Kerstin Weidner

**Affiliations:** Department of Psychotherapy and Psychosomatic Medicine, Faculty of Medicine and University Hospital Carl Gustav Carus, TUD Dresden University of Technology, Fetscherstr. 74, Dresden, 01307 Germany

**Keywords:** Post-COVID-19 condition, Psychological symptoms, Mental disorders, Psychopathology, Anxiety/ affective/ somatoform/ personality disorders, Psychosomatic/ post-COVID-19 outpatients, Patient health questionnaire (PHQ)

## Abstract

**Background:**

About 3–6% of individuals infected with SARS-CoV-2 develop post-COVID-19 condition (post-COVID), also known as multisystem disease, which comprises somatic and psychological symptoms. Nevertheless, there is an absence of research findings that differentiate patients with post-COVID from those with psychosomatic problems, for instance with regard to the presence of mental disorders. In addition, a comprehensive understanding of the factors that influence the manifestation of psychological symptoms in these patients is crucial for the development of multimodal interventions and lead to a destigmatization of post-COVID.

**Methods:**

A cross-sectional study was conducted at a Clinic for Psychotherapy and Psychosomatics (Dresden University Hospital) to compare the self-reported sociodemographic, clinical, and psychopathological characteristics of post-COVID patients (*n* = 357), attending a psychosomatic post-COVID outpatient clinic, with those of a group of patients with different psychosomatic disorders, attending a general psychosomatic outpatient clinic (*n* = 991). Clinical diagnoses were assessed by clinical experts according to the International Classification of Diseases (ICD-10), somatic symptom severity, depressive symptoms and anxiety, using the Patient Health Questionnaire (PHQ-D) (primary outcomes). In addition, in the post-COVID group, the influence of sociodemographic, clinical, and psychopathological variables on primary outcomes and incapacity to work were analyzed. The statistical methods encompassed linear and logistic regression analyses, generalized linear models, Mann–Whitney U-tests and chi-squared tests.

**Results:**

Post-COVID patients demonstrated a greater degree of PHQ-15 somatic symptom severity (B = 1.37, *p* < .001) and higher levels of somatic and psychological multimorbidity (OR = 1.14, *p* = .005). At the level of specific disorders, post-COVID patients demonstrated an elevated risk of somatoform disorders (OR = 3.25, *p* < .001), while the risk of anxiety disorders (OR = .40, *p* < .001), affective disorders (OR = .52, *p* < .001) and personality disorders (OR = .11, *p* < .001) was reduced. In post-COVID, female gender (*p* = .003), somatic and psychological multimorbidity (*p* ≤ .025), experience of psychotherapy (*p* ≤ .031), and stress (*p* < .001) were associated with increased psychological symptom burden.

**Conclusions:**

Post-COVID patients can be regarded as a specific group, distinguishable from psychosomatic patients and presumably with prevailing somatoform processing mechanisms. In accordance with the recommendations of the German post-COVID guideline, mental disorders should be accorded serious consideration in the treatment of post-COVID, with the objective of averting the risk of chronicity through early intervention.

**Trial registration:**

The present study does not report results of a health care intervention on human participants. Therefore no registration was necessary.

**Supplementary Information:**

The online version contains supplementary material available at 10.1007/s44192-026-00475-8.

## Background

The infection of the human respiratory tract with the Severe Acute Respiratory Syndrome (SARS) coronavirus (SARS-CoV-2) has been shown to result in a spectrum of symptoms, ranging from mild to severe, including fever, cough, fatigue, and dyspnoea. The most common of these symptoms are collectively known as Coronavirus Disease 2019 (COVID-19) [1]. The World Health Organization (WHO), has defined post-COVID-19 syndrome or condition (hereafter referred to as ‘post-COVID’) as symptoms that persist for at least three months or appear for the first time after a probable or confirmed SARS-CoV-2 infection. A diagnosis can only be made if the symptoms are not explained by any other condition [2]. A meta-analysis of 659,454 patients revealed that 50.1% reported at least one symptom between one and twelve months after SARS-CoV-2 infection [3]. Another meta-analysis, based on 31 studies involving predominantly hospitalized patients, reported a post-COVID prevalence of 32% between 90 and 120 days post-infection [4]. Taking into account the varying sample compositions and timeframes of epidemiological studies, the current German post-COVID guideline estimates prevalence rates to be lower, ranging from 3% to 6% [5, 6].

Patients infected with SARS-CoV-2 report more than 200 different symptoms, suggesting the involvement of multiple organs, including the skin, kidneys, gastrointestinal tract, endocrine, pulmonary, reproductive and cardiovascular systems. Neurological and psychological sequelae have also been documented among the most prevalent symptoms following a COVID-19 infection [7]. The most frequently reported psychological symptoms are fatigue (28.3%), cognitive/ memory impairment (19.7%)/ (17.5%), depression (18.3%), anxiety (16.2%), and sleep disturbances (13.5%). The most prevalent somatic symptoms are dyspnoea (24.0%), gastrointestinal symptoms (16.5%), cough (13.3%), joint pain (11.0%), palpitations (9.9%), and hair loss (8.4%) (for a systematic review and meta-analysis: 3).

A cluster analysis of 30 specific symptoms in a sample of 12,053 patients up to one year after acute SARS-CoV-2 infection revealed fatigue, neurocognitive disorders and anxiety/depression to be among the five most prevalent [8]. Studies involving patients at least three months after SARS-CoV-2 infection reported prevalence rates for clinically relevant depression (8–32%), anxiety (19–34%), post-traumatic stress disorder (PTSD, 9–18%), cognitive disorders (19–29%) [9–12], and somatic stress disorder (21–66%), respectively [13–15]. However, the prevalence rates are preliminary and inconsistent, due to the reliance on self-report questionnaires, the lack of control groups and retrospective data assessment [16–18]. Evidence has shown that SARS-CoV-2 infection is associated with an increased risk of mental disorders [19]. One study included a control group of patients suffering from respiratory tract diseases. The latter reported an increased incidence of affective-, anxiety-, and psychotic disorders, as well as brain fog, in patients after SARS-CoV-2 infection [18]. However, while the differences in mood and anxiety disorders ceased after 417–457 days, those related to cognitive deficits (‘brain fog’) and psychotic disorders persisted at the end of the two-year follow-up period. This led to the conclusion that transient symptom trajectories exist, and that contracting the virus is associated with a small but not negligible increase in the absolute risk of developing a mental disorder [19].

Findings reported the impact of several variables on the evolvement of post-COVID. Prevalence rates were lower among vaccinated individuals [20–22] and after infection with Omicron BA.1 or BA.2 virus variants [18, 20]. Risk factors for developing post-COVID include female gender, older age, lower socioeconomic status, belonging to an ethnic minority, obesity, smoking, existing mental or somatic comorbidities (e.g. anxiety disorders and/or depression, asthma, diabetes, immunosuppression, coronary heart disease) or hospitalization [22, 23]. Moreover, the time since infection has been identified as influencing factor, with symptoms significantly reduced one year after infection [24–26]. Similar risk factors with respect to psychological symptoms [e.g. depressive symptoms, anxiety and somatic symptoms, somatoform disorders [27] ] have been identified. These include female gender [28, 29], a history of mental disorders or psychotherapeutic, psychosomatic, or psychiatric treatment, the perceived severity of the acute infection [30, 31], obesity [32], smoking, and chronic physical illnesses [33].

However, the pathogenesis of post-COVID is currently considered to be incompletely understood [34–36]. The above-mentioned influencing variables support the assumption of an interaction between psychological and biological factors in the pathogenesis of both psychological and somatic symptoms of post-COVID [17, 37]. It is a heterogeneous entity with likely many subtypes, each of which is subject to different risk factors and pathophysiological mechanisms [26, 34, 38]. Potential pathophysiological mechanisms include persistent or permanent organ damage caused by COVID-19 or persistent SARS-CoV-2 infection, as well as autoimmunity or immune dysregulation. Other mechanisms involve pro-inflammatory cytokines that increase blood-brain barrier permeability and neurogenesis in the limbic system, vascular disease or coagulopathy, and reactivation of latent viruses, such as the Epstein-Barr virus [7, 35–37, 39]. However, psychological distress in patients with post-COVID symptoms may result from a significant deterioration in their quality of life and ability to perform daily activities [39, 40]. Additionally, psychological stressors such as social isolation, lockdown measures, and uncertainty about the future may increase vulnerability to mental disorders [37], which may, in turn, be responsible for the persistence of post-COVID symptoms. Furthermore, disadvantageous subjective interpretations of pre-existing symptoms might also play a role and contribute to a higher mental burden of post-COVID.

Currently, no clear biomarkers or causal therapy are available, but a significant influence of (pre-existing) psychological factors has been demonstrated [6]. Moreover, due to the increased psychological morbidity, affected individuals may be exposed to stigmatization as being mentally ill, which can lead to inadequate therapies. Research findings that differentiate post-COVID patients from those with psychosomatic problems are lacking, particularly with regard to the presence of mental disorders, sociodemographic, clinical, and psychological variables. It is unclear whether post-COVID patients differ from those with primary mental health diagnoses and somatic comorbidities when validated self-report measures and clinical psychological diagnostic assessments are used.

In the present paper, we aimed to examine the question whether post-COVID patients differ from patients with mental disorders without post-COVID symptoms (but with somatic comorbidities) in terms of mental diagnoses and psychological symptoms, sociodemographic factors, and certain clinical variables (primary aim). Furthermore, we aimed to investigate whether sociodemographic (e.g. age, gender, partnership, education), psychological (e.g., a history of psychotherapy, psychological multimorbidity), behavioral (smoking, alcohol consumption) and clinical variables (e.g. body mass index, hospitalization, time since last COVID-19 infection or vaccination, number of COVID-19 infections, COVID-19 vaccination, somatic multimorbidity) influence the intensity of depression and somatic symptoms (secondary aim), as well as the occurrence of anxiety and incapacity to work in post-COVID patients (tertiary aim). The present results may contribute to the destigmatization of post-COVID patients and may help to address their special needs by developing multimodal treatment programmes.

## Methods

### Participants

Patients were enrolled who presented at the psychosomatic post-COVID outpatient clinic of the Department of Psychotherapy and Psychosomatic Medicine at the University Hospital of the Dresden University of Technology, between 1 June 2021 and 30 November 2023. The post-COVID group was compared to a control group of patients who presented at the general psychosomatic outpatient clinic during the same period. At the psychosomatic post-COVID outpatient clinic, patients with ongoing or exacerbated symptoms after a COVID-19 infection were offered diagnostical assessment and advise for further diagnostic and treatment options. Patients at the general psychosomatic outpatient clinic presented with complex physical-mental conditions (also referred to as patients with psychosomatic disorders/ problems), diverse mental health problems and mental disorders and, in case of clinically relevant psychopathology, were offered specialized outpatient or inpatient treatment.

The recruiting institution is a clinic for psychosomatic medicine, which in Germany constitutes a distinct medical specialty alongside psychiatry. Accordingly, the patient population systematically differs from that of a primarily psychiatric institution. Severe mental disorders such as schizophrenia or bipolar disorders are predominantly treated in psychiatric clinics and are therefore typically underrepresented in psychosomatic samples. At the same time, the treatment spectrum of a psychosomatic clinic encompasses a wide range of patients with underlying somatic conditions and comorbid mental disorders. These include, among others, functional and chronic conditions such as irritable bowel syndrome, polycystic ovary syndrome, or migraine, as well as conditions such as takotsubo cardiomyopathy, endometriosis, cardiac arrhythmias, spinal diseases, or neurological diseases, each accompanied by concurrent mental comorbidity.

Inclusion criteria for both study groups were the following: minimum age of 18 years, sufficient knowledge of German, capacity to consent, and completeness of the questionnaire diagnostics and ICD-diagnoses. In addition, in the post-COVID group, only patients with a clinical diagnosis of SARS-CoV-2 infection in their medical history (positive PCR test result or positive rapid test result) and the additional ICD-diagnosis U09.9 (post-COVID condition, unspecified) in their doctor´s letter (received after presentation at our post-COVID outpatient clinic) were included.

Exclusion criteria for both groups: severe forms of intellectual impairment or mental disability, acute psychotic symptoms, acute suicidal tendencies, and explicit refusal to allow the questionnaire data to be used for research purposes. In the control group, the presence of the additional ICD-diagnosis U09.9 was an exclusion criterion. Furthermore, patients from the control group were not enrolled when they reported the new onset or exacerbation of symptoms after a COVID-19 infection.

**Fig. 1:**
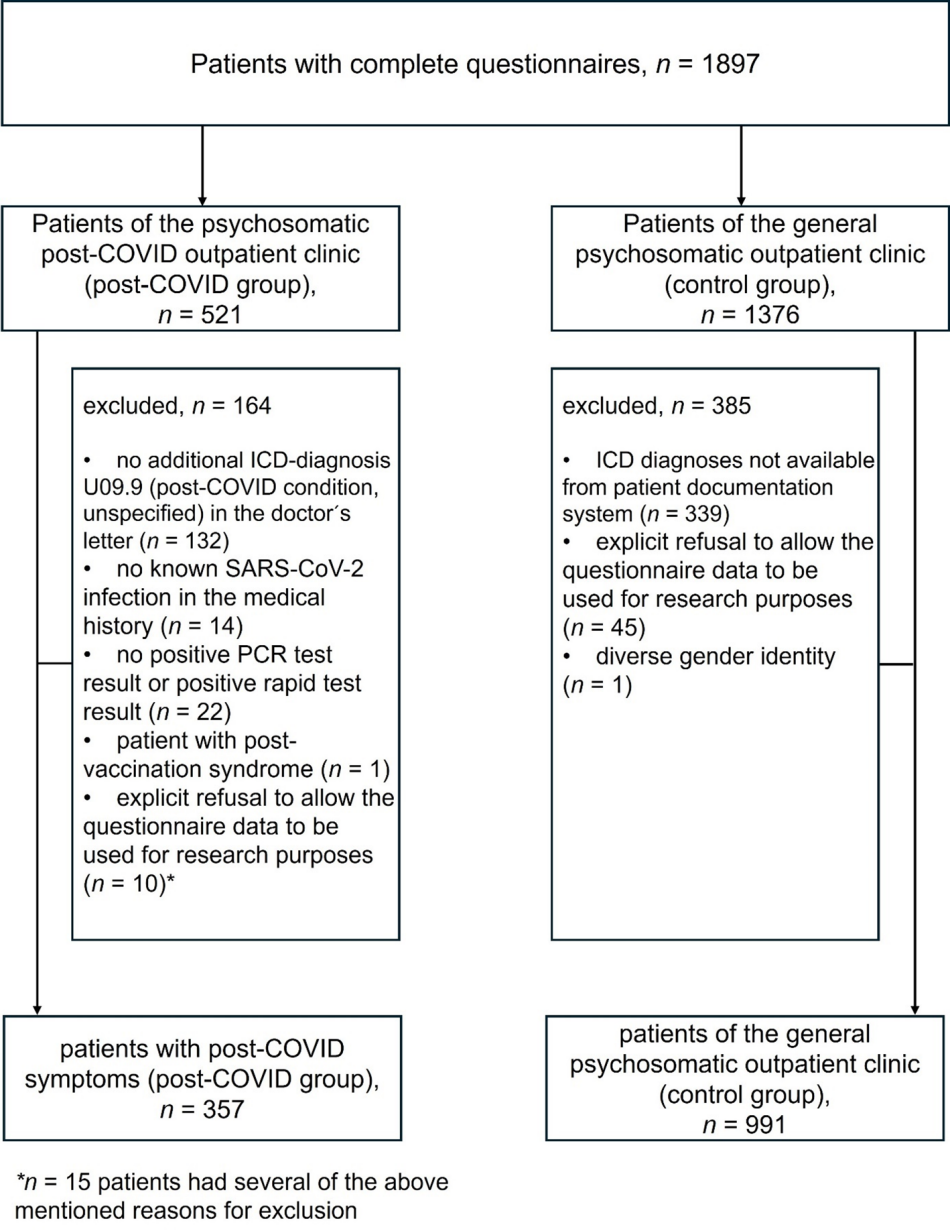
Displayed is the patient flow chart, including patients of the psychosomatic post-COVID outpatient clinic (post-COVID patients) and of the general psychosomatic outpatient clinic (control group) (time period between June 2021 to November 2023)

The patient flow chart is shown in Fig. 1. During the study period, *N* = 1897 patients completed the questionnaire, without missing values. For statistical reasons, one patient with a gender identity of diverse was excluded, as a group size of one person does not allow for statistical inference. After taking all inclusion and exclusion criteria into account, 357 patients were included in the post-COVID group (female: 67.2%; age: 47.5; SD: 11.4). *N* = 991 patients from the general psychosomatic outpatient clinic formed the control group (female: 69.6%; age: 36.6; SD: 13.9). Post-COVID patients reported an average of 1.2 previous infections with SARS-CoV-2. 80.4% of patients reported one infection, 18.2% reported two infections, and 1.4% reported three infections at the time of presentation. On average, 427.3 (SD = 217.3) days elapsed between the infection associated with the onset of post-COVID symptoms and the initial consultation at the post-COVID outpatient clinic.

Data on vaccination status was available for *n* = 229 post-COVID patients (64.1%). At the time of COVID-19 infection, *n* = 134 patients were unvaccinated, *n* = 4 patients reported incomplete vaccination protection from one vaccination, and *n* = 91 patients had complete vaccination protection from at least two vaccinations. Information on hospitalization status was available for *n* = 352 patients (98.6%). In *n* = 32 patients, COVID-19 required hospitalization, of whom *n* = 18 patients required ventilation.

## Procedure and measures

During an initial outpatient psychosomatic-psychotherapeutic consultation, the patient’s medical history was assessed, clinical psychological diagnostics were performed, and questionnaire diagnostics were conducted. According to good clinical practice guidelines, patients were offered treatment tailored to their specific symptoms, which primarily included psychoeducation, an explanation of the disorder model, and recommendations for further diagnostic and therapeutic options, such as symptom-specific interventions (e.g. physiotherapy, ergotherapy), dietary advice, smell training, medication or psychopharmacotherapy.

Measures: The extent of psychopathology was assessed by a self-report measure, the Patient Health Questionnaire-D (PHQ-D) [40, 41]. Further anamnestic, psychopathological and sociodemographic data were collected via a questionnaire (see Supplementary Material S1). In addition, an assessment of mental disorders by experienced clinical psychologists and physicians specialized in psychosomatic medicine was realized under supervision of a senior consultant (during a multiprofessional case conference), using the International Diagnosis Checklists for ICD-10 (IDCL) [42]. The analyzes included primary and secondary diagnoses which were further specified as ‘secured’ or ‘suspected’. Diagnoses specified as ‘Excluded’ or ‘Condition after’ were not taken into account.

The *PHQ-D* [40, 41] checks the diagnostic criteria of the Diagnostic and Statistical Manual DSM-IV [43] and includes modules for recording somatic symptom severity, depressive symptoms, anxiety and eating disorders as well as alcohol abuse. It also includes questions on psychosocial functioning, stressors, critical life events and, for women, menstruation, pregnancy and childbirth. It comprises 78 items with 2- to 5-point response scales, depending on the module. A dimensional evaluation is possible in the somatic symptom severity (PHQ-15), depressive symptoms (PHQ-9) and stress (psychosocial stress factors) modules. Threshold values are available for the modules assessing somatic symptom severity (PHQ-15) and depressive symptoms (PHQ-9). The severity levels for PHQ-15/ PHQ-9 are minimal (0 to < 5), mild (5 to < 10), moderate (10 to < 15), and for PHQ-15 severe (15 to 30); for PHQ-9 marked (15 to < 20) and severe (20 to 27) [44]. For the diagnosis of mental disorders (Axis I), sensitivity is 85%, specificity is 70%. Internal consistency for the somatic symptom severity module (PHQ-15) is Cronbach’s α = .79, and for the depressive symptoms module (PHQ-9) is Cronbach’s α = .88 [45]. In the sample studied, Cronbach’s α is α = .77 for the PHQ-15 and α = .84 for the PHQ-9.

The somatic and psychological multimorbidity of patients was operationalized as the sum of the 14 categories of illnesses (e.g. diseases of the nervous system and mental disorders, diseases of the cardiovascular system), specified in a questionnaire (see Supplementary Material S1). Body Mass Index (BMI) classification was defined according to WHO guidelines as follows [46]: underweight: BMI < 18.5; normal weight: BMI 18.5 to < 25; overweight: 25 < BMI < 30; obese: BMI ≥ 30.

Further information such as time of presentation, hospitalization as an indirect marker of COVID-19 disease severity, number of COVID-19 infections and vaccination status (time since last vaccination, fully vaccinated/no vaccination) were taken from the patients’ medical reports after their initial presentation at the psychosomatic post-COVID outpatient clinic and evaluated in conjunction with the questionnaire data.

## Statistical analyses

Chi-square tests or Fisher´s exact test (the latter in case of expected frequency of a category of less than five) were used to compare the post-COVID and control group in terms of the frequency of categorical sociodemographic characteristics, somatic symptom severity (PHQ-15) and depressive symptoms (PHQ-9), and categorical health variables (e.g. ICD-10- diagnoses, somatic comorbidities). Post hoc tests were performed using adjusted standardized residuals [47] to identify specific differences in multicategorical variables. Bonferroni corrections were applied in the post hoc tests to address alpha error accumulation. The adjusted significance levels resulting from the Bonferroni corrections are listed in Supplementary Material S2 or are mentioned in the results section.

To compare self-reported somatic and psychological comorbidities between groups, age, gender, and educational attainment were controlled for as confounding variables in logistic regression models. Due to the small number of patients who reported infectious diseases (*n* = 34), no logistic regression model was calculated for this variable. To compare the ICD-10 diagnoses of mental and behavioral disorders (F diagnoses) and somatic symptom severity between the groups, the confounding variables such as age, gender, educational attainment, and somatic and psychological multimorbidity were included in logistic regression models.

A simple linear regression model was used to compare the post-COVID and control group in terms of age. Multiple linear regression analyses were performed to compare the two groups in terms of the dependent variables somatic symptom severity (PHQ-15), depressive symptoms (PHQ-9) and stress (PHQ-D). A logistic regression analysis was performed to compare the dependent variable panic syndrome or other anxiety syndromes (PHQ-D). Age, gender, school leaving certificate (as an indicator of socioeconomic status) and somatic and psychological multimorbidity were included in the models as confounding variables, as they are each associated with both post-COVID and psychopathological measures [22, 48–51].

Since the two patient groups differed significantly in age, all group comparisons were repeated with and without adjusting for age to estimate the impact of age on effect sizes (Supplementary Material S3).

Furthermore, in a first step, simple linear and logistic regression analyses were performed with each individual predictor [age, gender, hospitalization, previous psychotherapeutic treatment, smoking status, somatic and psychological multimorbidity, relationship status, educational attainment, BMI, stress (PHQ-D), vaccination status, time since last COVID-19 vaccination, time since last COVID-19 infection, number of COVID-19 infections, and alcohol syndrome (PHQ-D)] on the dependent variable (metric: PHQ-9/ 15, dichotomous: anxiety syndromes, incapacity to work). In a second step, the significant predictors were included in joint multiple linear or logistic regression models. Due to the high number of missing values in the variables vaccination status (37.0%) and time between vaccination and admission (89.6%), many patients could not be included in the regression models. Following, the regression analyses for predicting the variables depressive symptoms, somatic symptom severity, panic syndrome or other anxiety syndromes and incapacity to work were performed without including the vaccination variables. Additionally, we reanalyzed the vaccination status data using multiple imputation. To accomplish this, we applied the Markov-Chain Monte Carlo (MCMC) algorithm with a maximum of ten iterations and Predictive Mean Matching (PMM). We defined the variables gender, age, educational attainment, and number of comorbidities as predictors for modeling vaccination status.

If the assumption of normally distributed residuals was violated when linear regression models were calculated, bootstrapping was applied with 1000 samples and BCa (bias corrected and accelerated) confidence intervals. When implementing the logistic regression models, the requirement for a linear relationship with the logit transformation of the dependent variable was violated in some models for age. In this case, age was divided into quartiles and included in the models in its classified form. To optimize the classification performance of the models, cut-off values were optimized using ROC (receiver operator characteristic) curves and the Youden index. The significance level was set at α $\:\le\:$. 05. The statistical analyzes were performed using IBM SPSS Statistics 29.0.2.0.

## Results

### Comparisons of the study samples with respect to sociodemographic variables

Post-COVID patients were significantly older than the psychosomatic comparison patients (B = 10.86; t(1346) = 13.25; *p* < .001; BCa 95% CI [9.47, 12.23]). The groups did not differ significantly in terms of gender (χ² [1] = .71, *p* = .401). Compared to the control group, post-COVID patients were significantly more likely to be in a partnership (χ² [1] = 27.18, *p* < .001) and significantly less likely to live alone (χ² [1] = 15.29, *p* < .001). Participants in the post-COVID group were significantly better educated than the control group. Post-COVID patients were more likely to have completed a secondary school leaving certificate, while they were significantly less likely to have completed a lower secondary school leaving certificate than the control group (χ² [1] = 11.01, *p* < .001). In addition, post-COVID patients were significantly more likely to have completed an apprenticeship (χ² [1] = 17.02, *p* < .001), while patients in the control group were significantly more likely to have no vocational qualification (χ² [1] = 66.03, *p* < .001). Post-COVID patients were significantly more likely to be employed (χ² [1] = 36.21, *p* < .001), while patients in the control group were significantly more likely to be in training or studying (χ² [1] = 32.74, *p* < .001) or unemployed (χ² [1] = 6.66, *p* = .010). The differences between post-COVID patients and the psychosomatic control sample are presented in Table 1.

**Table 1:** Sociodemographic characteristics compared between post-COVID patients (post-COVID group) and patients attending the general psychosomatic outpatient clinic (control group)

		Post-COVID outpatients (*n* = 357)		Psychosomatic outpatients (*n* = 991)		*p* ^b^	
		*n* ^a^	%	*n* ^a^	%		
Age (*n* = 1348)	mean (SD)	47.47	(11.36)	36.61	(13.91)	**< .001** ^**c**^	
Gender (*n* = 1348)	Female	240	67.2	690	69.6	.401	
	Male	117	32.8	301	30.4		
Housing situation (*n* = 1348)	Living alone	69	19.3	298	30.1	**< .001** ^**c**^	
	With partner/ children/friends	286	80.1	680	68.6	**< .001** ^**c**^	
	Nursing home/ residential home	2	.6	9	.9	.531	
	No permanent residence	0	.0	4	.4	.229	
Partnership (*n* = 1347)	Stable partnership	272	76.4	605	61.0	**< .001** ^**c**^	
	No stable partnership	84	23.6	386	39.0		
School leaving certificate (*n* = 1348)	Lower secondary school leaving certificate (Hauptschule)	13	3.6	90	9.1	**< .001** ^**c**^	
	Secondary school leaving certificate (10. Klasse)	183	51.3	430	43.4	.010	
	A-levels (Abitur)/ vocational baccalaureate (Fachabitur)	158	44.3	453	45.7	.636	
	No school leaving certificate	3	.8	18	1.8	.202	
Professional qualification (*n* = 1347)	Vocational training/ technical college	203	56.9	437	44.1	**< .001** ^**c**^	
	University of applied sciences/ University	126	35.3	289	29.2	.032	
	No professional qualification	14	3.9	231	23.3	**< .001** ^**c**^	
	Others	14	3.9	33	3.3	.604	
Employment situation (*n* = 1348)	Employed	265	74.2	556	56.1	**< .001** ^**c**^	
	Self-employed	20	5.6	54	5.4	.913	
	Unemployed	30	8.4	135	13.6	**.010** ^**c**^	
	Vocational training/studies	15	4.2	159	16.0	**< .001** ^**c**^	
	Early retirement/ Old-age pension/ Occupational disability	16	4.5	47	4.7	.841	
Ability to work (*n* = 1345)	Yes	164	45.9	648	65.6	**< .001** ^**c**^	
	No	193	54.1	340	34.4		
Disability/reduced earning capacity pension (*n* = 1347)	Temporary	4	1.1	13	1.3	.162	
	Permanent	8	2.2	23	2.3	.089	
Psychotherapeutic pre-treatments (*n* = 1347)	Outpatient	124	84.9	460	89.1	.163	
	Day clinic	10	6.8	89	17.2	**.002** ^**c**^	
	Inpatient	26	17.8	160	31.0	**.002** ^**c**^	

SD = standard deviation; ^a^for some values, *n* changes due to missing values; ^b^p-values of the chi-square tests; ^c^significant (for k = 1 individual test without Bonferroni corrections; for k > 1 individual tests after Bonferroni corrections); *k* = independent tests with a simple null hypothesis

## Comparison with respect to multimorbidity and severe somatic symptoms (PHQ-15)

Post-COVID patients showed significantly increased somatic and psychological multimorbidity compared to the control group, adjusted for age, gender and educational attainment (OR = 1.14, 95% CI [1.04, 1.25], *p* = .005).

Patients with post-COVID conditions more often reported cardiovascular - (χ² [1] = 46.90, *p* < .001), sensory organ - (χ² [1] = 19.10, *p* < .001), lung - (χ² [1] = 27.69, *p* < .001), gastroenterological - (χ² [1] = 5.89, *p* = .015), musculoskeletal - (χ² [1] = 52.94, *p* < .001) and infectious diseases (χ² [1] = 12.54, *p* < .001), as well as less often diseases of the nervous system and mental disorders (χ² [1] = 9.82, *p* = .002), than those in the control group. The differences remained after controlling for confounding variables (cardiovascular diseases: OR = 1.67, 95% CI [1.22, 2.28], *p* = .001; sensory organ diseases: OR = 1.57, 95% CI [1.07, 2.29], *p* = .020; lung diseases: OR = 2.03, 95% CI [1.43, 2.88], *p* < .001; musculoskeletal diseases: OR = 1.78, 95% CI [1.31, 2.43], *p* < .001). However, no significant differences were observed for gastroenterological diseases (OR = 1.10, 95% CI [.78, 1.54], *p* = .593). Compared to the control group, post-COVID patients were less likely to report diseases of the nervous system and mental disorders (OR = .67, 95% CI [.51, .87], *p* = .003) (see Supplementary Material S4).

In a logistic regression model adjusted for age, gender, educational attainment, somatic and psychological multimorbidity, post-COVID patients had a significantly increased risk of pain in the arms, legs or joints (OR = 2.78, 95% CI [2.09, 3.70], *p* < .001), headaches (OR = 1.87, 95% CI [1.40, 2.49], *p* < .001), chest pain (OR = 1.77, 95% CI [1.22, 2.57], *p* = .003), dizziness (OR = 1.62, 95% CI [1.19, 2.20], *p* = .002) and shortness of breath (OR = 3.71, 95% CI [2.74, 5.04], *p* < .001). In contrast, the chance of abdominal pain (OR = .58, 95% CI [.38, .88], *p* = .011), abdominal discomfort like nausea, flatulence or indigestion (OR = .53, 95% CI [.37, .74], *p* < .001) and pain or problems during sexual intercourse (OR = .56, 95% CI [.34, .94], *p* = .029), constipation, irritable bowel syndrome or diarrhoea (OR = .65, 95% CI [.45, .92], *p* = .015) was significantly reduced in the post-COVID group compared to the control group. The odds of severely debilitating back pain (OR = 1.13, 95% CI [.84, 1.51], *p* = .424), menstrual pain or problems (OR = .93, 95% CI [.57, 1.53], *p* = .784), fainting spells (OR = 1.76, 95% CI [.71, 4.38], *p* = .221), palpitation (OR = 1.02, 95% CI [.75, 1.40], *p* = .881), sleep disorders (OR = 1.13, 95% CI [.84, 1.53], *p* = .412) and fatigue or loss of energy (OR = 1.01, 95% CI [.75, 1.36], *p* = .958) were not significant beyond the control variables.

**Fig. 2:**
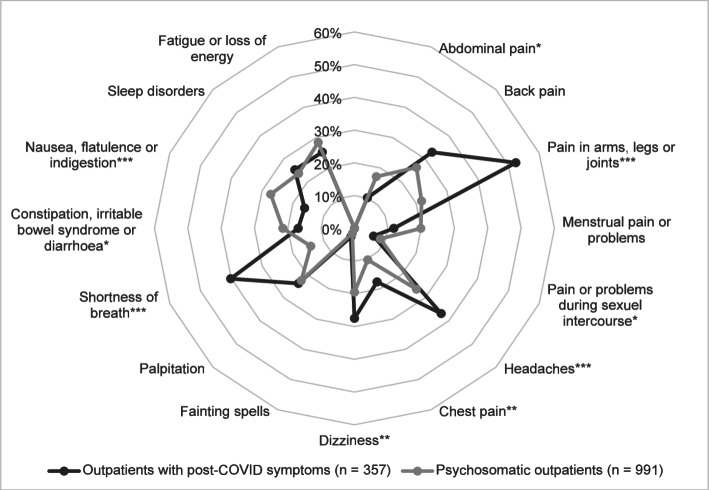
Severely impairing somatic symptoms as assessed in the Patient-Health-Questionnaire (PHQ-15) (Löwe et al., 2002) compared between post-COVID patients and psychosomatic outpatients (control group). (***p ≤ .001; **p ≤ .01; *p ≤ .05; only results at ***p ≤ .001 and **p ≤ .01 were significant after Bonferroni-correction)

After Bonferroni correction (p/ 15 ≤ .003), the reduced chance of abdominal pain, pain or problems during sexual intercourse and constipation, irritable bowel syndrome or diarrhoea were no longer significant. We compared the results with and without adjustment for age, and found that the latter were a lot (Delta OR ≥ .2) higher for pain in the arms, legs or joints and lower for headaches, menstrual pain or problems, and fainting spells. For an overview see Fig. 2 and Supplementary Material S3. 

## Comparison with respect to body mass index (BMI), smoking, alcohol consumption

In comparison with patients in the control group, post-COVID patients demonstrated a substantially diminished probability of being underweight (OR = .17, 95% CI [.05, .54], *p* = .003), even after controlling for age, gender, and education (Table 2).

Patients in the control group reported significantly more frequent cigarette consumption (t = 50.56, *p* < .001) and alcohol consumption (t = 6.16, *p* = .013). Although there was a significant difference in the number of cigarettes consumed (χ² = 10.59, *p* = .014), the latter was no longer significant after applying the Bonferroni correction (Table 2).

After adjusting for age, gender and education, post-COVID patients had a significantly reduced risk of developing an alcohol syndrome, compared to the control group (OR = .25, 95% CI [.10, .58], *p* = .001) (Table 2).

**Table 2:** *BMI*,* smoking and alcohol consumption were compared between post-COVID outpatients and general psychosomatic outpatients (control group).*

		Post-COVID-outpatients (*n* = 357)		Psychosomatic outpatients (*n* = 991)		*p* ^a^	*p* ^b^
		*n*	%	*n*	%		
Body-Mass-Index (*n* = 1343)	mean (SD)	26.74	5.44	25.27	6.39		
- underweight		3	.8	94	9.5	**< .001** ^**c**^	**< .003** ^**c**^
- normal weight		153	43.0	463	46.9	.194	
- overweight		112	31.5	246	24.9	.016	
- obesity		88	24.7	184	18.6	.016	
Smoking (yes/no; *n* = 1347)		39	10.9	296	29.9	**< .001** ^**c**^	
- < 10 cigarettes/week		12	30.8	42	14.2	.009	
- < 10 cigarettes/day		15	38.5	93	31.4	.368	
− 10 to 20 cigarettes/day		9	23.1	136	45.9	.007	
- > 20 cigarettes/day		3	7.7	25	8.4	.841	
Alcohol (yes/no; *n* = 1348)		151	42.3	495	49.9	**.013** ^**c**^	
− 1 to twice/month		61	40.4	218	44.0	.424	
− 1 to twice/week		70	46.4	178	36.0	.021	
− 3 to five times times/week		16	10.6	75	15.2	.162	
- daily		4	2.6	24	4.8	.230	
Alcohol syndrome (PHQ-D^d^; *n* = 1344)		6	1.7	91	9.2	**< .001** ^**c**^	**.001** ^**c**^

SD = standard deviation; ^a^p value Chi-square test; ^b^p value of the predictor of group membership (post-COVID vs. control group) in logistic regression; ^c^significant (for k = 1 individual tests without Bonferroni corrections; for k > 1 individual tests after Bonferroni corrections); k = independent tests with simple null hypothesis; ^d^Patient Health Questionnaire-D [40]

### Comparison with respect to mental disorders

No significant differences were observed between post-COVID patients and the control group in terms of either the number of mental disorders (U = 168,281.50, *p* = .064) or the number of comorbid mental disorders (U = 172,799.50, *p* = .292).

Compared to the psychosomatic control group, post-COVID patients had a significantly increased risk of somatoform disorders (OR = 3.25, 95% CI [2.47, 4.27], *p* < .001). Post-COVID patients were significantly less likely to suffer from affective disorders (OR = .52, 95% CI [.38, .71], *p* < .001), anxiety disorders (OR = .40, 95% CI [.25, .65], *p* < .001) or personality disorders (OR = .11, 95% CI [.03, .48], *p* = .003) than the control group (Table 3). These results remained significant after Bonferroni correction and controlling for age, gender and education. However, as there were no cases of eating disorders in the post-COVID group, the logistic regression model based on the data could not reliably estimate the association between group membership and eating disorders (OR = .000, *p* = .993).

**Table 3:** Diagnoses compared between post-COVID patients (post-COVID group) and patients from the general psychosomatic outpatient clinic (control group)

Disorder or disorder group (ICD code)	Post-COVID outpatients (*n* = 357)		Psychosomatic outpatients (*n* = 991)		*p* ^a^	*p* ^b^
	*n*	%	*N*	%		
Substance use disorders (F1)	6	1.7	28	2.8	.237	
Schizophrenia, schizotypal and delusional disorder (F2)	0	.0	7	.7	.200	
Affective disorders (F3)	72	20.2	307	31.0	< .001	**< .001** ^**c**^
- depressive episode	36	10.1	91	9.2		
- recurrent depressive disorder	34	9.5	198	20.0		
- dysthymia	0	.0	19	1.9		
- bipolar disorder	1	.3	2	.2		
Anxiety disorders (F40, F41)	23	6.4	147	14.8	< .001	**< .001** ^**c**^
- panic disorder	13	3.6	50	5.0		
- agoraphobia	5	1.4	32	3.2		
- social phobia	0	.0	31	3.1		
- generalized anxiety disorder	1	.3	10	1.0		
- specific phobia	0	.0	20	2.0		
Obsessive-compulsive disorder (F42)	2	.6	21	2.1	.051	
Reactions to severe stress and adjustment disorders (F43)	40	11.2	130	13.1	.350	
- posttraumatic stress disorder	4	1.1	32	3.2		
- adjustment disorder	35	9.8	95	9.6		
Somatoform disorder (F45)	204	57.1	222	22.4	< .001	**< .001** ^**c**^
- somatization disorder	2	.6	4	.4		
- undifferentiated somatization disorder	175	49.0	112	11.3		
- hypochondriacal disorder	1	.3	7	.7		
- somatoform autonomic dysfunction	9	2.5	30	3.0		
- persistent pain disorder	24	6.7	71	7.2		
Eating disorders (F50)	0	.0	125	12.6	< .001	
Personality disorders (F60)	2	.6	70	7.1	< .001	**< .001** ^**c**^

^a^p-value of the chi-squared test; ^b^p-value of the predictor of group membership (post-COVID vs. the control group) in logistic regression; ^c^significant (for k = 1, individual tests without Bonferroni corrections; for k > 1, individual tests after Bonferroni corrections); k = independent tests with a simple null hypothesis

### Comparison with respect to PHQ somatic symptom severity and depressive symptoms

In the multiple linear regression model, which controlled for age, gender, educational attainment, somatic and psychological multimorbidity, post-COVID patients exhibited significantly higher somatic symptom severity than the control group (B = 1.37, t(1326) = 4.2995% CI [.74, 1.99], *p* < .001). Depressive symptom scores (B = -1.32, t(1332) = -3.39, 95% CI [-2.08, − .55], *p* < .001) and stress scores (B = -1.96, t(1328) = -7.70, 95% CI [-2.46, -1.46], *p* < .001) were significantly lower in the post-COVID group than in the control group.

After Bonferroni correction, significantly more post-COVID patients had a severe degree of somatic symptom severity (χ² [1] = 9.61, *p* = .002), while significantly more patients in the control group reported a mild degree (χ² [1] = 19.36, *p* < .001). Conversely, with regard to depressive symptoms, patients in the control group reported severe symptoms significantly more often (χ² [1] = 23.04, *p* < .001), whereas patients in the post-COVID group reported mild or subthreshold symptoms significantly more often (χ² [1] = 23.04, *p* < .001) (Table 4).

The risk of panic or other anxiety syndromes was significantly lower for post-COVID patients compared to the control group, even after controlling for the above-mentioned confounding variables (OR = .50, 95% CI [.38, .67], *p* < .001).

**Table 4:** The symptom burden was compared between post-COVID patients (post-COVID group) and patients attending a general psychosomatic outpatient clinic (control group), using the Patient Health Questionnaire-D (PHQ-D)

		Post-COVID outpatients (*n* = 357)		Psychosomatic outpatients (*n* = 991)		*p* ^a^
		n	%	n	%	
Somatic symptom severity (*n* = 1334)	mean (SD)	13.14	(4.88)	11.60	(5.23)	< .001^b^
- minimal		15	4.3	78	7.9	.021
- mild		64	18.2	298	30.3	< .001^b^
- moderate		140	39.8	322	32.8	.016
- severe		133	37.8	284	28.9	.002^b^
Depressive symptoms (*n* = 1340)	mean (SD)	11.18	(5.14)	13.03	(6.28)	< .001^b^
- minimal		25	7.0	102	10.4	.072
- mild		125	35.2	218	22.1	< .001^b^
- moderate		110	31.0	250	25.4	.046
- marked		68	19.2	235	23.9	.072
- severe		27	7.6	180	18.3	< .001^b^
Stress (*n* = 1336)	mean (SD)	6.47	(3.64)	8.46	(3.96)	< .001^b^
Panic syndrome oder other anxiety syndrome (*n* = 1342)		90	25.3	406	41.2	< .001^b^

SD = standard deviation; ^a^ *p* values of the group predictors (post-COVID vs. control group) in the linear/logistic regression models and when comparing the severity levels of the chi-square tests; ^b^significant (for k = 1 individual tests without Bonferroni corrections; for k > 1 individual tests after Bonferroni corrections); k = independent tests with simple null hypothesis

### Predictors of depressive symptoms (in the post-COVID group)

Using bootstrapping, it was found that patients who had undergone prior psychotherapeutic treatment exhibited significantly higher levels of depressive symptoms than patients who had not undergone prior psychotherapeutic treatment (B = 2.20, t = 4.04, BCa 95% CI [1.22, 3.22], *p* ≤ .001). Somatic and psychological multimorbidity (B = .75, t = 5.44, BCa 95% CI [.48, 1.02], *p* ≤ .001) and stress (B = .77, t = 12.17, BCa 95% CI [.65, .90], p $\:\le\:$ .001) were positively correlated with the patients’ depressive symptoms.

A joint multiple linear regression model was then calculated using bootstrapping of the significant predictors of depressive symptoms (see Table 5). The model explained 33.6% of the variance in depressive symptoms. Taking into account the other predictors in the model, previous psychotherapeutic treatment (B = 1.06, t = 2.29, BCa 95% CI [.07, 1.95], *p* = .031), somatic and psychological multimorbidity (B = .48, t = 3.92, 95% CI [.26, .70], *p* < .001) and stress (B = .70, t = 10.98, BCa 95% CI [.55, .85], *p* < .001) continued to show significant positive correlations with depressive symptoms in post-COVID patients.

**Table 5:** Regression model for predicting depressive symptoms in post-COVID patients (post-COVID group), using the Patient Health Questionnaire-D (PHQ-D)^a^

BCa-95%-confidence interval^b, c^						
Predictors	B	β	SF^b^	*p* ^b^	Lower limit	Upper limit
Psychotherapeutic pre-treatment	1.06	.10	.46	**.031**	.07	1.95
Multimorbidity	.48	.18	.11	**< .001**	.26	.70
Stress (PHQ-D)	.70	.49	.07	**< .001**	.55	.85
Constant	5.02		.54	**< .001**	4.07	5.98
R^2^	.34					
corrected R^2^	.34					
*F*(df = 3; 347)	60.14			**< .001**		

^a^*n* = 351; ^b^estimated by bootstrapping with 1000 samples; ^c^Confidence interval type: Bias-corrected and accelerated (BCa) method; B = non-standardized regression coefficient; β = standardized regression coefficient; SF = standard error;

### Predictors of somatic symptom severity (in the post-COVID group)

In the group of post-COVID patients, women (B = 1.92; t = 3.53, 95% CI [.85, 2.99], *p* ≤ .001), patients with prior psychotherapeutic treatment (B = 1.96, t = 3.76, 95% CI [.94, 2.99], *p* ≤ .001), patients with increased somatic and psychological multimorbidity (B = .96, t = 7.59, 95% CI [.71, 1.21], *p* ≤ .001) and patients with increased stress (B = .44, t = 6.51, 95% CI [.31, .58], *p* < .001) showed a significantly increased somatic symptom severity. The multiple regression model of significant predictors explained 23.2% of the variance in somatic symptom severity (see Table 6). Taking into account the other predictors in the model, female gender (B = -1.46, t = 2.95, 95% CI [-2.43, − .49], *p* = .003), somatic and psychological multimorbidity (B = .81, t = 6.50, 95% [.56, 1.05], *p* < .001) and stress (B = .32, t = 4.95, 95% CI [.19, .45], *p* < .001) continued to show significant correlations with somatic symptom severity in the post-COVID group. However, the correlation between previous psychotherapeutic treatment (B = .83, t = 1.72, 95% CI [-.12, 1.75], *p* = .086) was not significant when the other predictors were taken into account.

**Table 6:** Regression model for predicting somatic symptom severity in post-COVID patients (post-COVID group), using the Patient Health Questionnaire-D (PHQ-D)^a^

95%-Confidence interval						
Predictors	B	β	SF	*P*	Lower limit	Upper limit
Gender	-1.46	− .14	.50	**.003**	-2.43	− .49
Psychotherapeutic pre-treatment	.83	.08	.49	.086	− .12	1.75
Multimorbidity	.81	.31	.12	**< .001**	.56	1.05
Stress (PHQ-D)	.32	.24	.07	**< .001**	.19	.45
Constant	9.12		.57	**< .001**	7.99	10.24
R^2^	.24					
corrected R^2^	.23					
*F*(df = 4; 346)	27.43			**< .001**		

^a^*n* = 351; B = non-standardized regression coefficient; β = standardized regression coefficient; SF = standard error

### Predictors of anxiety

Using simple logistic regression models, a significantly increased chance of anxiety syndrome was found in post-COVID patients who had undergone prior psychotherapeutic treatment (OR = 2.36, 95% CI [1.45, 3.85], *p* < .001). In addition, the likelihood of anxiety syndrome was significantly increased in cases of elevated somatic and psychological multimorbidity (OR = 1.24, 95% CI [1.10, 1.40], *p* < .001) and elevated stress (OR = 1.24, 95% CI [1.16, 1.34], *p* < .001). Subsequently, a logistic regression model of the significant predictors was established (see Supplementary Material S5). The model showed a variance explanation of 20.2%. Previous psychotherapeutic treatment (Wald $\:{\chi\:}^{2}$(1) = 6.22, 95% CI [1.15, 3.28], *p* = .013), somatic and psychological multimorbidity (Wald $\:{\chi\:}^{2}$(1) = 5.03, OR = 1.94, 95% CI [1.02, 1.33], *p* = .025) and stress (Wald $\:{\chi\:}^{2}$(1) = 26.36, OR = 1.13, 95% CI [1.13, 1.31], *p* < .001) also showed a significant explanatory contribution to the presence of an anxiety syndrome in post-COVID in the joint model.

### Predictors of incapacity to work

The prediction of incapacity to work at the time of initial contact was explored. Using logistic regression analyses, only the time between COVID-19 infection and initial contact in days showed a significant association with incapacity to work ($\:{\chi\:}^{2}$(1) = 9.41, *p* = .002, *n* = 342). The risk among patients with post-COVID decreased by .2% per day since the COVID-19 infection (OR = .998, *p* = .003, 95% CI [.997, .999]).

### Impact of vaccination- exploratory analyses

Among the 225 patients with post-COVID for whom data on vaccination status (fully vaccinated versus not vaccinated) was available, no association was found between vaccination status and depressive symptoms, somatic symptom severity, anxiety syndromes, or incapacity to work. Simple linear and logistic regression models were not significant, neither for depressive symptoms (F(1, 221) = .09, *p* = .763, *n* = 223), somatic symptom severity (F(1, 219) = .46, *p* = .498, *n* = 221), anxiety syndromes (χ² [1] = 1.31, *p* = .253, *n* = 224) nor for incapacity to work (χ² [1] = 1.43, *p* = .232, *n* = 225). The results were reanalyzed using multiply imputed data for vaccination status, and did not change (depressive symptoms: F(1, 353) = .11, *p* = .746, *n* = 355; somatic symptom severity: F(1, 350) = 2.76, *p* = .097, *n* = 352; anxiety syndromes: χ² [1] = 2.57, *p* = .109, *n* = 356; incapacity to work: χ² [1] = .38, *p* = .538, *n* = 357).

## Discussion

A cross-sectional study was conducted to investigate psychopathological symptoms in post-COVID patients, compared to a psychosomatic control group, with both groups being enrolled in a psychosomatic outpatient clinic.

### Main differences in sociodemographic and clinical characteristics

As part of the first research question, post-COVID patients were compared with those in the control group in terms of socio-demographic and socio-medical characteristics, debilitating physical symptoms, and somatic and psychological comorbidities. On average, post-COVID patients were older than those in the control group. This finding is consistent with previous studies showing that older adults are at an increased risk of post-COVID symptoms [22], while mental disorders are significantly more prevalent in younger individuals [49, 50].

In line with previous findings on the gender distribution in post-COVID condition and mental disorders, the proportion of women in both the post-COVID group and the control group is significantly higher than the proportion of men [22, 49, 50]. Further differences, which may primarily reflect the age difference between the groups, were found in living arrangements, relationship status, educational and professional qualifications, and employment status. In addition, post-COVID patients were significantly more likely to report being unable to work than those in the control group. Mental and behavioral disorders were the second most common reason for incapacity to work in Germany in 2021, accounting for a total of 17.7% of all days. Although we did not evaluate the cause of work incapacity, our results emphasize the significant limitations and economic impact of post-COVID symptoms [52, 53].

In the present study, a high proportion (84.9%) of post-COVID patients had undergone outpatient psychotherapy prior to their diagnosis. Though the present study assessed prior psychotherapeutic treatment in a simplistic manner, it may serve as an indirect marker for pre-existing mental disorders or, at least, an increased psychological vulnerability. In line with the latest research, (pre-)existing mental disorders are now recognized as a replicated risk factor for post-COVID conditions [22, 54–56]. However, the proportion of prior psychotherapeutic treatment may also be higher in the psychosomatic care facility studied, as positive previous experiences with psychotherapy may increase the likelihood of seeking psychosomatic treatment again.

### Somatic symptom burden (PHQ-15) and possible explanations

Compared to the control group, the post-COVID group reported a broader spectrum of burdensome physical symptoms, including pain in the extremities or joints, headaches, chest pain, dizziness, and shortness of breath, which are frequently observed in post-COVID [3, 35] but are also recognized in other clinical contexts [57, 58]. In contrast, patients in the control group more often reported specific symptoms originating in the gastrointestinal tract, such as abdominal pain, nausea, flatulence or indigestion, constipation, irritable bowel syndrome or diarrhoea. In the present study, the level of somatic symptom burden was assessed using the PHQ-15. This self-report questionnaire reflects the extent of subjective impairment due to physical symptoms, without allowing conclusions regarding their underlying genesis. Accordingly, post-COVID patients exhibited a significantly higher somatic symptom burden on average compared with the control group. Overall, 77.6% of post-COVID patients reported moderate to severe impairment due to physical symptoms.

From a multifactorial perspective, these findings can be interpreted as the result of an interplay of multiple contributing factors. In addition to currently discussed biomedical mechanisms, such as viral persistence, autoimmune processes, or alterations of the microbiome—which so far do not provide a conclusive explanation—empirical evidence indicates that psychosocial and cognitive processes are also associated with the intensity and persistence of symptom burden [7, 34–36]. Both the present data and previous studies suggest that heightened attentional focus on bodily sensations, interpreting them as threatening, as well as processes such as catastrophizing and fear-avoidance, may be associated with increased symptom burden and functional limitations [59, 60]. The results of the present study suggest that post-COVID patients in our sample constitute a heterogeneous group, displaying a wide range of burdensome somatic symptoms that may be dysfunctionally processed through selective attention and misinterpretation, thereby contributing to a vicious cycle of avoidance or endurance [13].

### Further findings with respect to the PHQ

In the present study, the severity of depressive symptoms and somatic symptom severity in the post-COVID group is comparable to earlier studies [13, 71, 72]. The proportion of patients with post-COVID exhibiting clinically relevant depressive symptoms, as measured by the PHQ-9, is descriptively 5.6-fold higher compared to the general population [73]. The patients in the control group, on the other hand, showed significantly higher levels of depressive symptoms and stress. The latter finding may be attributed to the fact that the PHQ-stress measure assesses various stress domains, including sexual issues, relationship conflict, caregiving, and work/school stress. These domains may be less relevant for post-COVID patients. However, these patients appear to experience higher stress levels due to health-related concerns (data not shown).

### ICD mental disorders, somatoform disorders and diagnostics, conceptual limitations

In line with previous findings [13–15, 61], post-COVID patients in our sample were more likely to have somatoform disorders than patients in the psychosomatic control group. The latter group more often suffered from affective and anxiety disorders alongside burdensome gastrointestinal symptoms. This finding is consistent with evidence indicating that gastrointestinal symptoms are common in affective and anxiety disorders [62].

On closer inspection, the observed differences with respect to somatoform disorders may be explained primarily by the significantly higher proportion of patients with undifferentiated somatization disorder in the post-COVID group (49.0%), while other somatoform disorders, such as somatization disorder or somatoform autonomic dysfunction, were distributed more or less equally between the groups. The high rate of undifferentiated somatization disorders observed in the present study is significantly higher than the rates reported in two previous studies of patients with post-COVID with respect to somatic stress disorders, which were 21.3% and 25.6% [13, 15], but lower than the 66.0% reported in a third study [14]. The variation in rates may be attributed to differences in assessment methodologies. Lower rates were found when a structured clinical interview was applied [13], whereas higher rates resulted when cut-off scores based on self-report questionnaires were used [14, 15].

Psychologists and physicians specializing in psychosomatic medicine made a diagnosis in the area of somatoform disorders if the criteria were met. First, the physical symptoms presented by the patients could not be completely explained by medical findings. Secondly, additional psychosocial stress factors could be identified (e.g. financial problems, social isolation, health anxiety, pain catastrophizing or selective attention to bodily processes) that may contribute to the development and maintenance of somatic symptoms in post-COVID. This approach is consistent with a bio-psycho-social model of disease [63].

At the time of admission to the psychosomatic post-COVID outpatient clinic, a comprehensive review of patients’ medical histories and examination findings was conducted. Standard medical diagnostic procedures and laboratory tests revealed no medical findings in the majority of patients. In individual cases, findings such as vitamin D deficiency were observed, but these did not fully explain the symptoms.

The use of somatoform diagnoses according to ICD-10 carries conceptual limitations. The diagnostic criterion of insufficient or unexplained medical findings poses a particular challenge for conditions with still incompletely understood pathogenesis, such as post-COVID. Diagnostic classification is therefore necessarily bound to the current state of biomedical knowledge and may change as scientific understanding evolves. The biomedical pathogenesis of post-COVID condition still remains incompletely understood. Therefore, the ICD-10 somatoform classifications are insufficient and carry the risk of stigmatizing post-COVID patients by implying a psychogenic etiology. Diagnoses reported here should be interpreted in the context of the classification criteria and knowledge available at the time of assessment. In this regard, newer classification systems, such as bodily distress disorder (ICD-11), offer conceptual advantages, as they focus more on psychological criteria—including cognitive, emotional, and behavioral responses to physical symptoms—rather than primarily on their medical unexplainedness [64, 65].

As mentioned above, in line with an expanded understanding of post-COVID, a multifactorial model integrating biological, psychological, and social factors is increasingly applied. Alongside the discussed biomedical mechanisms, there is empirical evidence that psychosocial and cognitive processes can contribute to the development and maintenance of symptoms [63, 66].

Patients in the post-COVID group were significantly less likely to meet the criteria for affective, anxiety, eating, and personality disorders than those in the control group. Consequently, patients in the latter group exhibited a wider range of mental health conditions than those with post-COVID symptoms, for whom somatoform disorders appear to represent the predominant condition. When comparing the disorder rates of the post-COVID group with those of the general German population, it also becomes apparent that affective disorders were descriptively more frequent, with a 2.1-fold increase, while anxiety disorders occurred significantly less frequently [49, 50]. The high proportion of anxiety disorders in the general population is particularly influenced by the high proportion of specific phobias, which occurred only rarely in both the post-COVID and the control group. This suggests that patients with specific phobias who require treatment rarely seek treatment in the setting studied. Nevertheless, the low rate of anxiety disorders seems to contradict earlier studies that showed a significantly increased risk of depressive and anxiety disorders following a SARS-CoV-2 infection [69, 70]. These disorders may also contribute to the development of post-COVID symptoms.

In line with our findings of a high prevalence of somatoform disorders, a growing number of studies indicate that psychosocial stressors may influence the development and persistence of post-COVID [56]. In a prospective cohort study, anxiety symptoms, depressive symptoms, concerns about COVID-19, subjective stress, and loneliness prior to COVID-19 illness were associated with an increased risk of post-COVID, defined as COVID-19 symptoms lasting at least four weeks, regardless of sociodemographic factors, health behaviors and somatic comorbidities [67]. In another study, psychosomatic symptom burden (e.g. my COVID symptoms ‘scare me’ or are ‘signs of a serious illness’) at the time of COVID-19 illness predicted the risk of somatic post-COVID symptoms and the extent of symptom-related impairment from symptoms six months after illness, independent of medical parameters such as BMI or COVID-19 severity [68]. Post-COVID patients also showed significantly higher levels of pain catastrophizing and ‘fear avoidance’ (avoiding exercise due to fear of worsening symptoms) compared to a healthy control group, which may contribute to the persistence of somatic symptoms [59].

#### Influencing variables on psychological outcomes

Stress and somatic and psychological multimorbidity were each positively correlated with depressive symptoms, somatic symptom severity, and anxiety syndromes, in patients with post-COVID. Furthermore, patients who had previously undergone psychotherapeutic treatment exhibited higher levels of depressive symptoms and anxiety, but not somatic symptom severity. Previous studies have found that prior psychotherapeutic treatment is associated with depressive and anxiety symptoms, as well as somatic symptom severity, following a diagnosis of SARS-CoV-2 [37, 74]. Although previous studies have found that women have an increased risk of depressive symptoms, somatic symptoms, and anxiety symptoms after contracting COVID-19, women in the present sample showed an increased risk of somatic symptom severity only [28, 29, 74]. Consequently, general practitioners may be less likely to refer patients with high somatic symptom burdens to physicians who specialize in psychosomatic medicine. This can contribute to chronic conditions and unintended negative effects on mental health.

In a subsequent exploratory analysis, we found no impact of vaccination on primary psychological outcome measures. This finding seems to contradict existing evidence. For example, a prospective cohort study found that individuals who were not vaccinated against SARS-CoV-2 were 85% more likely to receive a mental health diagnosis one year after infection. Meanwhile, those who were vaccinated were 9% less likely to receive a mental health diagnosis [75]. However, in the post-COVID sample, information on the vaccination status of 35.9% of patients was unavailable, which may have biased the estimate. Therefore, our null finding regarding the impact of the vaccination on psychological outcomes should be treated with the utmost caution and is presumably due to the small sample size. Reanalyzes with multiply imputed data also did not find an impact of vaccination status. However, the results hint at a reduced symptom burden in vaccinated patients with respect to somatic symptom severity. When investigating the impact of vaccination on psychological outcomes, the time since vaccination should also be considered. In the present sample, patients were vaccinated at a median of nearly twelve months prior to enrollment. Previous studies have shown that receiving a vaccine for SARS-CoV-2 more than twelve months prior is associated with long-term symptoms post-infection [76].

### Strengths and limitations, and outlook for future research

As part of the study, a large sample of post-COVID patients from a naturalistic care setting at a psychosomatic outpatient clinic specializing in post-COVID conditions was examined for somatic impairments and psychopathological symptoms. Unlike earlier studies, which often relied solely on self-reported measures for post-COVID diagnosis, this study included only patients in the post-COVID group who had received a clinical diagnosis of post-COVID from medical and psychological experts specialized in psychosomatic medicine.

The present study is cross-sectional, which means that it does not allow for causal reasoning. Patients’ self-reports of somatic or psychological comorbidities, as well as previous psychotherapeutic treatments, may have been influenced by their experience with the infection. On average, patients commenced admission to our post-COVID outpatient unit 427 days after infection. Data on physical and psychological comorbidities do not allow us to conclude whether these occurred before or as a result of SARS-CoV-2 infection. Additionally, these variables were assessed using self-report measures. Therefore, physical and psychological multimorbidity as predictors of symptom intensity should be interpreted with considerable caution. To improve the reliability and objectivity of the measurements, future studies should collect data on somatic comorbidities based on medical histories, and use structured interviews to diagnose life-time and current mental disorders. Therefore, the assessment of psychotherapeutic pre-treatment as an indirect measure of mental disorders should be replaced by a thorough structured diagnostic assessment.

The classification of somatoform disorders according to ICD-10 in the context of post-COVID can be viewed critically. ICD-10 highlights medically unexplained physical symptoms as a central feature of this group of disorders, whereby a diagnosis is also permitted if somatic disorders are present but do not explain ‘the nature and extent of the symptoms, the suffering and the internal involvement’ of the patient [77]. However, post-COVID is a new phenomenon characterized by a variety of somatic complaints, as demonstrated by the results of this study, yet its pathogenesis remains unclear [7, 34, 35]. Somatic damage caused by SARS-CoV-2 that leads to long-term consequences may not yet be measurable with currently available diagnostic methods, or the interaction of already identified biomedical anomalies (e.g. viral persistence, autoimmunity or changes in the microbiome) may not yet be sufficiently understood [35]. A focus on medically unexplained physical symptoms, as required by the ICD-10, should therefore be critically reconsidered in the case of post-COVID, as this implicitly suggests a predominantly psychosocial origin for which there is no scientific evidence.

The current classification systems for mental disorders, the DSM-5 and the ICD-11, no longer require that somatic symptoms be medically unexplained. More suitable diagnostic alternatives, which should be used in future research instead of somatoform disorders according to ICD-10, are somatic symptom disorder in DSM-5 and bodily distress disorder in ICD-11. The latter came into force in 2022 but has not yet been introduced for morbidity coding. Both can be diagnosed regardless of whether somatic disorders are present [64, 65].

A major limitation of the present study is the possible presence of selection bias. The patients were recruited from a psychosomatic care facility, which may have appealed more to post-COVID patients with mental health issues than post-COVID outpatient clinics that specialize in areas such as neurology or infectious diseases. Additionally, patients with more severe post-COVID symptoms who had undergone prior treatment may be disproportionately represented. Both factors may have led to an overestimation of the rates of psychopathological consequences associated with COVID-19. Therefore, our results are limited to the context of psychosomatic studies. Generalizability to the general population or to patients from other specialized outpatient clinics for post-COVID conditions is limited.

Another major issue is the potential for selection bias in the control group due to the naturalistic study setting. The general psychosomatic outpatient group was significantly younger than the post-COVID group (average age of 36.6 years versus 47.5 years). This fundamental age difference may have affected how symptoms were presented and interpreted. We addressed this issue by statistically controlling all group comparisons for age. Furthermore, we compared the results with and without adjusting for age. Most results changed slightly, and some effect sizes increased when age was controlled for. Thus, the age differences may have led to an underestimation of the effect sizes. However, future research should carefully match groups by age.

Another limitation is the lack of information regarding the control group’s exposure to SARS-CoV-2-related variables. Patients attending a general psychosomatic outpatient clinic may also have been affected by post-COVID symptoms or probable symptom worsening after COVID-19. However, when assigning patients to specific outpatient clinics (psychosomatic post-COVID vs. general psychosomatic), care was taken to ensure that patients at the general psychosomatic clinic did not have primary concerns relating to the virus. While the time course of psychological symptoms could be specified for the post-COVID group, no information on the time course of psychological symptoms for the control group was accessible. Therefore, the results may have been biased by differing symptom durations.

Another limiting point worth mentioning is the choice of the study period. The study period spans different virus variants, pre- and post-vaccination and treatment availability, lockdown and post-lockdown phases. The present study, which is rather small and cross-sectional, did not control for these dynamic phases during the pandemic. Future studies should control for historical confounders or compare earlier and later phases of the pandemic. In the present study, we statistically controlled for some variables (e.g., vaccination status and time since last SARS-CoV-2 infection or vaccination), but we could not systematically assess this information in our naturalistic setting, which resulted in missing data.

Further prospective studies should be conducted that take both biomedical and psychosocial factors into account to investigate their differential contributions to the development of persistent symptoms following COVID-19. Additionally, future studies should investigate the prevalence of somatic stress disorders in patients with post-COVID using structured clinical interviews in prospective cohort studies based on general population samples at multiple study sites. Controlled prevention studies aimed at reducing the risk of post-COVID complications by modifying psychological processes (e.g. addressing health anxiety or modifying selective attention, and catastrophizing) could also provide important insights into the contribution of psychological factors. Future studies should examine the effectiveness of psychotherapeutic interventions for various post-COVID symptom clusters in randomized controlled trials [8, 26].

## Conclusions

Post-COVID condition is a heterogeneous, multisystem disease that follows SARS-CoV-2 infection. It is characterized by a broad spectrum of burdensome mental and physical symptoms [7, 34, 78]. It significantly impacts patients´ quality of life, work capacity and healthcare systems [6, 79]. This cross-sectional study compared post-COVID patients from a specialized outpatient clinic with a psychosomatic control group from a general outpatient clinic, in terms of sociodemographic, clinical, and psychopathological variables. Post-COVID patients exhibited a high level of comorbidity with somatoform and depressive disorders, as well as experiencing severe impairment due to somatic symptoms and increased somatic and psychological multimorbidity. The data analyzed suggest that patients with somatic and mental comorbidities, a history of psychotherapeutic treatment, and psychosocial stress factors may be at a higher risk of developing psychopathologies in the context of post-COVID. The impairment caused by somatic symptoms was more pronounced in women, in cases of increased somatic and psychological multimorbidity, and in cases of higher levels of stress. Patients with somatic and psychological comorbidities, a history of psychotherapy, and increased stress exhibited more severe depressive symptoms, somatic symptoms, and had an increased risk of developing an anxiety syndrome. In accordance with the current post-COVID-19 guidelines in Germany, the findings underscore the importance of recognizing and addressing psychological impairments as a key component of post-COVID-19 treatment. The rationale underpinning this approach is to facilitate early intervention aimed at modifying maladaptive processing of somatic symptoms and thereby mitigating the risk of prolonged sequelae [6, 13]. Prospective longitudinal studies should investigate the differential contributions of biological, psychological and social factors to the development and maintenance of post-COVID.

## Supplementary Information

Below is the link to the electronic supplementary material.

Supplementary Material 1

## Data Availability

The data and materials on which the conclusions of the paper rely can be obtained from the corresponding author upon reasonable request.

## Abbreviations

BMI: Body mass index
Coronavirus Disease 2019: Covid-19
DSM: Diagnostic and statistical manual
ICD-10: International classification of diseases
OR: Odds ratio
post-COVID-19 syndrome: Post-coronavirus disease 2019 syndrome
PCR: Polymerase Chain reaction
PMM: Predictive mean matching
SARS-CoV-2 virus: Severe acute respiratory syndrome coronavirus 2
WHO: World health organization
