# Supplementary material for: Psychopathologies in post-COVID outpatients differ from a psychosomatic control sample in a cross-sectional study
Source: Discov Ment Health. 2026 May 20;6(1):90. doi: 10.1007/s44192-026-00475-8 (PMC13190902; doi:10.1007/s44192-026-00475-8)
Supplement: Supplementary file 1 — Supplementary Material 1 [file 44192_2026_475_MOESM1_ESM.docx]

Supplementary Material S1

**Questionnaire - Medical History**

Dear patient, we have a few questions for you below. Your answers will help us to classify your symptoms and enable us to offer you the appropriate treatment. We therefore ask you to answer all questions. **You can tick several answers if applicable.** We will ask you any clarifying or additional questions during the initial diagnostic consultation.

**If applicable, what illness(es) are you currently suffering from?**

**If applicable, please try to classify your current illness(es)!**

|  | Diseases of the nervous system and mental disorders  (e.g. MS – multiple sclerosis, epilepsy, ALS – amyotrophic lateral sclerosis, sleep disorders, epilepsy, migraine, meningitis, encephalitis, spinal muscular atrophy, Huntington's disease, depression, reaction to severe stress, adjustment disorder, anxiety disorder, obsessive-compulsive disorder, alcoholism, drug or medication addiction, schizophrenia) |
| --- | --- |
|  | Diseases of the cardiovascular system  (e.g. Blood clotting disorder, anaemia, diseases of the spleen, diseases of the leukocytes, polycythaemia, acute rheumatic fever, chronic rheumatic heart disease, hypertension [high blood pressure], heart disease, cerebrovascular disease, diseases of the arteries, arterioles and capillaries, diseases of the veins, lymph vessels and lymph nodes) |
|  | Endocrine and metabolic disorders  (e.g. thyroid gland, endocrine disorders, diabetes mellitus, pancreatic disease, malnutrition, vitamin/mineral deficiency, obesity) |
|  | Rheumatic diseases  (e.g. rheumatoid arthritis, ankylosing spondylitis) |
|  | Diseases of the sensory organs: eyes, throat, nose, ears  (e.g. glaucoma, visual disturbances and blindness, diseases of the outer ear, middle ear and mastoid process, inner ear) |
|  | Diseases of the lung  (e.g. chronic asthma, COPD – chronic obstructive pulmonary disease, tuberculosis) |
|  | Gastroenterological diseases  (e.g. chronic gastritis, gastroesophageal reflux, chronic  inflammatory bowel disease, irritable bowel syndrome – possibly ‘psychosomatic’, any disease of the liver) |
|  | Skin diseases  (e.g. infections of the skin and subcutaneous tissue, bullous dermatoses, dermatitis  and eczema, papulosquamous skin diseases, urticaria and erythema, diseases of the skin and subcutaneous tissue caused by radiation exposure, diseases of the skin appendages) |
|  | Musculoskeletal diseases  (e.g. arthropathies such as osteoarthritis, systemic connective tissue diseases, diseases of the spine and back, diseases of the soft tissues, osteopathies and chondropathies) |

|  | Complications during pregnancy, childbirth and the postpartum period  (e.g. oedema, hypertension, bleeding in early pregnancy, excessive vomiting, nutritional problems, venous disorders, diabetes mellitus, miscarriage, complications during anaesthesia, premature birth) |
| --- | --- |
|  | Diseases of the urinary and reproductive systems  (e.g. glomerular diseases, tubulointerstitial kidney diseases, renal insufficiency, kidney stones or bladder stones, diseases of the kidney, ureter and urinary system, diseases of the male genital organs and mammary glands [breast glands], inflammatory diseases of the female pelvic organs, non-inflammatory diseases of the female genital tract such as endometriosis) |
|  | Cancers |
|  | Infectious diseases  (e.g. hepatitis, AIDS, gastroenteritis, cholera, salmonella infection, bacterial dysentery, amoebiasis, tuberculosis, syphilis, gonococcus, herpes, chlamydia, yaws, acute poliomyelitis, viral encephalitis, Creutzfeldt-Jakob disease, mycosis, candidiasis) |
|  | Congenital malformations, deformities and chromosomal abnormalities  (e.g. congenital malformations of the nervous system such as hydrocephalus and microcephaly, of the eye, ear, face and neck, of the circulatory system, respiratory system, digestive system, genital organs, urinary system, cleft lip, jaw and palate, malformations and deformities of the musculoskeletal system, Chromosomal abnormalities) |
|  | Other  What category of illness is it? |

**Do you have any of the symptoms listed below?**

|  | depressive symptoms (depression, loss of motivation, joy and interest, lack of energy, etc.) |
| --- | --- |
|  | anxiety symptoms (e.g. fear of certain situations, sudden palpitations, shortness of breath, feeling of loss of control, etc.) |
|  | unexplained physical symptoms, i.e. for which no organic cause has been found |
|  | nightmares, distressing memories, jumpiness, constant feeling of numbness, etc. |
|  | difficulties related to eating |
|  | difficulties related to addictive substances (e.g. alcohol, drugs, medication) |
|  | difficulties in relationships with other people |
|  | personality disorders |
|  | other complaints: |

**Have you been diagnosed with a neurological or mental illness by a doctor or psychotherapist?**

|  | No |
| --- | --- |
|  | Yes |

**If yes: Which neurological or mental illnesses have been diagnosed?**

|  | epilepsy |
| --- | --- |
|  | paralysis |
|  | depression |
|  | anxiety disorders |
|  | somatoform disorders |
|  | dissociative disorders |
|  | eating disorders |
|  | personality disorders |
|  | other: |

**Have you ever had surgery in your life?**

|  | No |  | Yes |
| --- | --- | --- | --- |
|  |  | Which parts of your body were operated on? | |
|  |  |  | head, neck |
|  |  |  | chest |
|  |  |  | abdominal cavity |
|  |  |  | limbs |
|  |  |  | skin |

**Do you take medication regularly?**

|  | No |  | Yes |
| --- | --- | --- | --- |
|  |  | Which medication(s) do you take regularly? | |
|  |  |  | analgesics |
|  |  |  | sleeping medication |
|  |  |  | antihypertensive |
|  |  |  | cholesterol-lowering agent |
|  |  |  | insulin |
|  |  |  | thyroid medication |
|  |  |  | laxative |
|  |  |  | psychopharmaceutical |
|  |  |  | contraceptive (e.g. pill, patch, coil) |
|  |  |  | other: |

**Have you been under medical treatment in the last 4 weeks?**

|  | No |  | Yes |
| --- | --- | --- | --- |
|  |  | With whom? | |
|  |  |  | with a specialist doctor |
|  |  |  | at a general practitioner's |

**Do you have any previous experience with psychotherapeutic methods?**

|  | No |  | Yes |
| --- | --- | --- | --- |
|  |  | What previous experience do you have with psychotherapy? | |
|  |  |  | outpatient psychotherapy |
|  |  |  | inpatient psychotherapy |
|  |  |  | day hospital |
|  |  |  | as part of hospital or rehabilitation treatment |

**Are you currently on sick leave from a doctor?**

|  | No |  | Yes |
| --- | --- | --- | --- |

**How many days were you unable to work last year (approximately)?** If you were not unable to work last year, please enter the number 0 here.

days

**What is your current employment status?**

|  | employed |
| --- | --- |
|  | self-employed |
|  | unemployed |
|  | in vocational training/ studying |
|  | in retraining/ further training |
|  | housewife/ househusband |
|  | early retirement / old-age pension / occupational disability |
|  | other: |

**Has a disability pension/reduced pension been applied for?**

|  | No |
| --- | --- |
|  | Yes, currently |
|  | Yes, at an earlier time |

**Has a disability pension/reduced pension been approved?**

|  | No |
| --- | --- |
|  | Yes, temporary |
|  | Yes, permanent |

**Is there a care level?**

|  | No |  | Yes |
| --- | --- | --- | --- |

**Do you have a severely disabled person's pass?**

|  | No |  | Yes |
| --- | --- | --- | --- |

**Do you smoke?**

|  | No |  | Yes |
| --- | --- | --- | --- |
|  |  | How much do you smoke? | |
|  |  |  | less than 10 cigarettes per week |
|  |  |  | less than 10 cigarettes per day |
|  |  |  | 10 to 20 cigarettes per day |
|  |  |  | more than 20 cigarettes per day |

**Do you drink alcohol?**

|  | No |  | Yes |
| --- | --- | --- | --- |
|  |  | How often do you drink alcohol? | |
|  |  |  | once or twice a month |
|  |  |  | once or twice a week |
|  |  |  | 3 to 5 times a week |
|  |  |  | daily |

**Please tell us your age, weight and height.**

| Age (in years) |  |
| --- | --- |
| Weight (in kg) |  |
| Height (in cm) |  |

**Sociodemographics**

Please give us a brief overview of yourself and your social environment by answering the following questions!

| **What is your current living situation?** | |
| --- | --- |
|  | live alone |
|  | live with partner/children/friends |
|  | live in a nursing home, residential home, etc. |
|  | have no permanent residence |
| **Are you in a committed relationship?** | |
|  | no fixed partnership |
|  | stable relationship |
| **Do you have children?** | |
|  | no children |
|  | one child |
|  | two children |
|  | three children |
|  | four or more children |
| **What is your highest level of education?** | |
|  | (to date) no school leaving certificate |
|  | special school certification/ secondary school leaving certificate/ graduation after the 9^th^ grade |
|  | secondary School leaving certificate/ Polytechnic secondary school leaving certificate/ completion after the 10^th^ grade |
|  | A-levels/vocational A-levels |
| **What is your highest professional qualification?** | |
|  | (to date) no vocational qualification |
|  | vocational training/ technical college |
|  | university of applied sciences/university |
|  | other |
| **How often have you sought medical/psychotherapeutic care in the last 12 months due to physical or mental health problems?** | |
|  | not once |
|  | once |
|  | twice |
|  | not three times |
|  | more than three times |
| **Nationality** | |
|  | German |
|  | rest of Europe* |
|  | outside Europe* |
|  | *Please indicate your nationality: |

**Supplementary Material S2**

*Adjusted alpha error levels of the study variables after Bonferroni correction*

| Variable | Number of individual tests | Adjusted alpha error level |
| --- | --- | --- |
| Housing situation | 8 | .00625 |
| School leaving certificate | 8 | .00625 |
| Professional qualification | 8 | .00625 |
| Employment situation | 5 | .01 |
| Disability/reduced earning capacity pension | 3 | .0167 |
| Psychotherapeutic pre-treatments | 3 | .0167 |
| Diseases of the cardiovascular system | 2 | .025 |
| Diseases of the sensory organs | 2 | .025 |
| Diseases of the lung | 2 | .025 |
| Musculoskeletal diseases | 2 | .025 |
| Gastroenterological diseases | 2 | .025 |
| Diseases of the nervous system and mental disorders | 2 | .025 |
| Pain in arms, legs or joints (PHQ-15) | 2 | .025 |
| Headaches (PHQ-15) | 2 | .025 |
| Chest pain (PHQ-15) | 2 | .025 |
| Dizziness | 2 | .025 |
| Shortness of breath | 2 | .025 |
| Abdominal pain | 2 | .025 |
| Nausea, flatulence or indigestion | 2 | .025 |
| Back pain | 2 | .025 |
| Menstrual pain or problems | 2 | .025 |
| Weight classifications based on BMI | 8 | .00625 |
| Number of cigarettes smoked | 8 | .00625 |
| Amount of alcohol drunk | 8 | .00625 |
| Underweight (logistic regression model) | 2 | .025 |
| Alcohol syndrome (PHQ-D) | 2 | .025 |
| Affective disorders | 2 | .025 |
| Anxiety disorders | 2 | .025 |
| Somatoform disorders | 2 | .025 |
| Personality disorders | 2 | .025 |
| Eating disorders | 2 | .025 |
| Somatic symptom severity (classification) (PHD-15) | 8 | .00625 |
| Depressive symptoms (classification) (PHQ-9) | 10 | .005 |

PHQ = Patient Health Questionnaire

**Supplementary S3**

**Table S3:** Group comparisons were made with and without adjusting for age. Patients with post-COVID symptoms have an increased risk if the OR is ≥ 1 and a decreased risk if the OR is < 1.

|  | **Adjusted for age** | **Not adjusted for age** |
| --- | --- | --- |
| somatic and psychological multimorbidity | OR = 1.14, 95% CI [1.04, 1.25], p = .005 | OR = 1.37, 95% CI [1.25, 1.50], p < .001 |
| cardiovascular diseases | OR = 1.67, 95% CI [1.22, 2.28], p = .001 | OR = 2.61. 95% CI [1.95, 3.50], p < .001 |
| sensory organ diseases | OR = 1.57, 95% CI [1.07, 2.29], p = .020 | OR = 2.27, 95% CI [1.58, 3.25], p < .001 |
| lung diseases | OR = 2.03, 95% CI [1.43, 2.88], p < .001 | OR = 2.42, 95% CI [1.74, 2.36], p < .001 |
| musculoskeletal diseases | OR = 1.78, 95% CI [1.31, 2.43], p < .001 | OR = 2.83, 95% CI [2.12, 3.77], p < .001 |
| diseases of the nervous system and mental disorders | OR = .67, 95% CI [.51, .87], p = .003 | OR = .67, 95% CI [.52, .86], p = .001 |
| pain in the arms, legs or joints (PHQ-15) | OR = 2.78, 95% CI [2.09, 3.70], p < .001 | OR = 3.49, 95% CI [2.66, 4.58], p < .001 |
| headaches (PHQ-15) | OR = 1.87, 95% CI [1.40, 2.49], p < .001 | OR = 1.59, 95% CI [1.21, 2.07], p < .001 |
| chest pain (PHQ-15) | OR = 1.77, 95% CI [1.22, 2.57], p = .003 | OR = 1.73, 95% CI [1.22, 2.47], p = .002 |
| dizziness (PHQ-15) | OR = 1.62, 95% CI [1.19, 2.20], p = .002 | OR = 1.52, 95% CI [1.14, 2.03], p = .005 |
| shortness of breath (PHQ-15) | OR = 3.71, 95% CI [2.74, 5.04], p < .001 | OR = 3.88, 95% CI [2.91, 5.18], p < .001 |
| abdominal pain (PHQ-15) | OR = 0.58, 95% CI [0.38, 0.88], p = .011 | OR = 0.48, 95% CI [0.32, 0.72], p < .001 |
| nausea, flatulence or indigestion (PHQ-15) | OR = 0.53, 95% CI [0.37, 0.74], p < .001 | OR = 0.44, 95% CI [0.32, 0.61], p < .001 |
| pain or problems during sexual intercourse (PHQ-15) | OR = 0.56, 95% CI [0.34, 0.94], p = .029 | OR = 0.60, 95% CI [0.36, 0.99], p = .044 |
| back pain (PHQ-15) | OR = 1.13, 95% CI [0.84, 1.51], p = .424 | OR = 1.23, 95% CI [0.93, 1.62], p = .153 |
| menstrual pain or problems (PHQ-15) | OR = 0.93, 95% CI [0.57, 1.53], p = .784 | OR = 0.58, 95% CI [0.37, .91], p = .017 |
| fainting spells (PHQ-15) | OR = 1.76, 95% CI [0.71, 4.38], p = .221 | OR = 1.52, 95% CI [0.65, 3.55], p = .330 |
| palpitation (PHQ-15) | OR = 1.02, 95% CI [0.75, 1.40], p = .881 | OR = .98, 95% CI [0.73, 1.32], p = .892 |
| constipation, irritable bowel syndrome or diarrhoea (PHQ-15) | OR = 0.65, 95% CI [0.45, 0.92], p = .015 | OR = 0.60, 95% CI [0.43, 0.84], p = .003 |
| sleep disorders (PHQ-15) | OR = 1.13, 95% CI [0.84, 1.53], p = .412 | OR = 1.09, 95% CI [0.81, 1.45], p = .579 |
| fatigue or loss of energy (PHQ-15) | OR = 1.01, 95% CI [0.75, 1.36], p = .958 | OR = .936, 95% CI [0.71, 1.24], p = .645 |
| Underweight | OR = .17, 95% CI [0.05, .54], p = .003 | OR = .09, 95% CI [0.03, .27], p < .001 |
| alcohol syndrome | OR = .25, 95% CI [0.10, 0.58], p = .001 | OR = .17, 95% CI [0.07, 0.39], p < .001 |
| somatoform disorders | OR = 3.25, 95% CI [2.47, 4.27], p < .001 | OR = 4.09, 95% CI [3.15, 5.32], p < .001 |
| affective disorders | OR = .52, 95% CI [0.38, 0.71], p < .001 | OR = .52, 95% CI [.38, .70], p < .001 |
| anxiety disorders | OR = .40, 95% CI [0.25, 0.65], p < .001 | OR = .41, 95% CI [0.26, 0.65], p < .001 |
| personality disorders | OR = .11, 95% CI [0.03, 0.48], p = .003 | OR = .08, 95% CI [0.02, 0.32], p < .001 |
| PHQ-15 | B = 1.37, t(1326) = 4.29, 95% CI [0.74, 1.99, p < .001] | B = 1.00; t(1327) = 3.28; 95% CI [0.40, 1.60], p = .001 |
| PHQ-9 | B = -1.32, t(1332) = -3.39, 95% CI [-2.08, -0.55], p < .001 | B = -2.08, t(1333) = -5.51, 95% CI [-2.82, -1.34], p < .001 |
| PHQ-stress | B = -1.96, t(1328) = -7.70, 95% CI [-2.46, -1.46], p < .001 | B = -2.14; t(1329) = -8.76, 95% CI [-2.61, -1.66], p < .001 |
| panic or other anxiety syndrome (PHQ) | OR = .50, 95% CI [0.38, 0.67], p < .001 | OR = .45, 95% CI [0.34, 0.59], p < .001 |

PHQ = Patient Health Questionnaire

**Supplementary Material S4**

**Figure S4:** Somatic and psychological comorbidities were compared between patients with post-COVID symptoms and psychosomatic outpatients (control group). (***p ≤ .001; **p ≤ .01; *p ≤ .05). After controlling for confounding variables, the difference for gastroenterological diseases disappeared, while the difference for sensory organ diseases remained significant at p = .020.


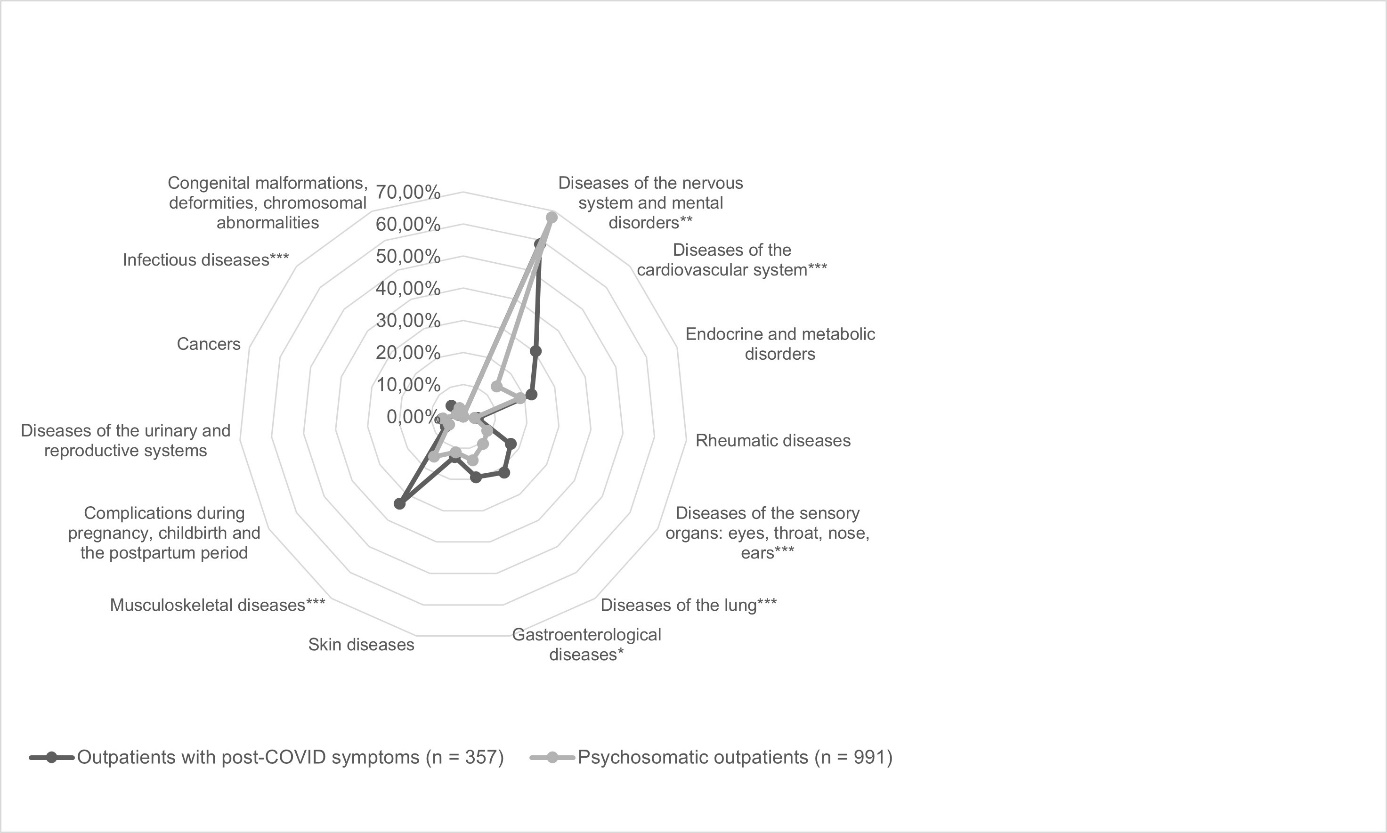


|  | Outpatients with post-COVID symptoms (n = 357) | Psychosomatic outpatients (n = 991) |
| --- | --- | --- |
| Diseases of the nervous system and mental disorders** | 58,80% | 68% |
| Diseases of the cardiovascular system*** | 30,50% | 14,10% |
| Endocrine and metabolic disorders | 22,40% | 18,60% |
| Rheumatic diseases | 4,50% | 3,60% |
| Diseases of the sensory organs: eyes, throat, nose, ears*** | 17,10% | 8,70% |
| Diseases of the lung*** | 21,60% | 10,50% |
| Gastroenterological diseases* | 19,30% | 13,90% |
| Skin diseases | 12,90% | 11,40% |
| Musculoskeletal diseases*** | 33,60% | 15,50% |
| Complications during pregnancy, childbirth and the postpartum period | 6,30% | 5,10% |
| Diseases of the urinary and reproductive systems | 6,70% | 6,40% |
| Cancers | 1,70% | 2,20% |
| Infectious diseases*** | 5% | 1,60% |
| Congenital malformations, deformities, chromosomal abnormalities | 2% | 2,90% |

**Supplementary Material S5**

**Table S5:** Regression model for predicting anxiety syndromes in patients with post-COVID symptoms (post-COVID group). using the Patient Health Questionnaire-D (PHQ-D)^a^

|  | | | |  | 95%-Confidence interval for OR | |
| --- | --- | --- | --- | --- | --- | --- |
| Predictors | B | SF | OR | *P* | Lower limit | Upper limit |
| Psychotherapeutic pretreatment | 0.66 | 0.27 | 1.94 | .013 | 1.15 | 3.28 |
| Multimorbidity | 0.15 | 0.07 | 1.17 | .025 | 1.02 | 1.33 |
| Stress (PHQ-D) | 0.12 | 0.04 | 1.22 | < .001 | 1.13 | 1.31 |
| Constants | -3.19 | 0.38 | 0.04 | < .001 |  |  |
| -2 Log-Likelihood | 347.88 |  |  |  |  |  |
| Nagelkerkes R² | 0.20 |  |  |  |  |  |
| $\boldsymbol{\chi}^{\boldsymbol{2}}$(1) | 51.74 |  |  | < .001 |  |  |
| ^a^*n* = 351; B = non-standardized regression coefficient; SF = standard error; OR = Odds Ratio | | | | | | |
